# Enhancing the Mechanical and Durability Properties of Fully Recycled Aggregate Concrete Using Carbonated Recycled Fine Aggregates

**DOI:** 10.3390/ma17081715

**Published:** 2024-04-09

**Authors:** Birori Jean, Hui Liu, Xudong Zhu, Xinjie Wang, Xiancui Yan, Tianyu Ma

**Affiliations:** 1 Department of Civil Engineering, Changzhou University, Changzhou 213164, China; jbirory1@gmail.com (B.J.); cczuzxd2020@163.com (X.Z.); wangxinjie@cczu.edu.cn (X.W.); yanxc@cczu.edu.cn (X.Y.); 20447120@smail.cczu.edu.cn (T.M.); 2 State Key Laboratory of Silicate Materials for Architectures, Wuhan University of Technology, Wuhan 430070, China

**Keywords:** carbonated recycled fine aggregates, fully recycled aggregate concrete, chloride permeability resistance, frost resistance

## Abstract

The global construction industry is increasingly utilizing concrete prepared from recycled aggregate as a substitute for natural aggregate. However, the subpar performance of recycled fine aggregate (RFA) has resulted in its underutilization, particularly in the structural concrete exposed to challenging environments, including those involving chlorine salts and freeze–thaw climates. This study aimed to enhance the performance of RFA as a substitute for river sand in concrete as well as fulfill the present demand for fine aggregates in the construction sector by utilizing accelerated carbonation treatment to create fully recycled aggregate concrete (FRAC) composed of 100% recycled coarse and fine aggregates. The impacts of incorporating carbonated recycled fine aggregate (C-RFA) at various replacement rates (0%, 25%, 50%, 75%, and 100%) on the mechanical and durability properties of FRAC were investigated. The results showed that the physical properties of C-RFA, including apparent density, water absorption, and crushing value, were enhanced compared to that of RFA. The compressive strength of C-RFC100 was 19.8% higher than that of C-RFC0, while the water absorption decreased by 14.6%. In a comparison of C-RFC0 and C-RFC100, the chloride permeability coefficients showed a 50% decrease, and the frost resistance increased by 27.6%. According to the findings, the mechanical and durability properties, the interfacial transition zones (ITZs), and micro-cracks of the C-RFC were considerably enhanced with an increased C-RFA content.

## 1. Introduction

Concrete has become the most popular material for non-structural and structural elements, and its manufacturing relies heavily on natural resources. However, with the rapid pace of new urbanization coupled with urban redevelopment and industrialization, a surge in the construction and demolition wastes (C&DWs) is produced. The uptick has led to escalating environmental concerns and an acute shortage of natural aggregate (NA) resources [1,2,3]. According to the statistics [4], the 21st century has experienced an extensive urbanization process, resulting in a substantial production of construction waste. Global urbanization rates were documented at 54.3% in 2016, and they climbed to 55% by 2018. Projections suggest a surge to 68% globally by the year 2050, leading to a steady rise in the generation of C&DWs [5]. Such vast quantities of C&DWs are frequently consigned to landfill-dumpsite or used as backfill for roadbeds. This mismanagement squanders resources, harms the environment, and contradicts the principles of eco-friendly growth [6]. This further contributes to significant ecological degradation, over-exploitation, and a severe scarcity of quality natural sands and gravels [7,8,9]. As such, the necessity for employing alternative raw materials in construction projects becomes paramount. In the quest for sustainability in the construction industry, which is based on the principle of resource conservation, the strategy of recycling C&DWs debris as a means of addressing the global strategic goal of reducing construction waste can have a significant beneficial impact on the environment and production costs [10,11,12,13]. The practice of utilizing C&DWs as recycled aggregate (RA) is gaining increasing traction worldwide.

Many researchers have dedicated their efforts to exploring the potential for reusing RA. However, it has been found that due to the poor quality of RA, its application remains markedly restricted in many countries, often limited to lower-value purposes such as constructing unbound roadways [14,15].

Particularly in the case of RA derived from waste concrete, the cracks generated by the machine crushing process and the presence of old mortar attached to the surface of RA impart poor properties such as low apparent density, high porosity, and high water adsorption, etc. [16]. In these circumstances, mixing fresh recycled aggregate concrete (RAC) requires more water than mixing natural aggregate concrete (NAC). Additionally, the interfacial transition zones (ITZs) between the new cement mortar and aggregate in RAC are typically inferior to those in NAC, owing to the presence of adhered old mortar. Consequently, the resulting hardened RAC exhibits compromised durability, diminished strength, and reduced elastic modulus [17,18,19,20]. Compared to recycled coarse aggregate (RCA), reports in the literature suggest that recycled fine aggregate (RFA) tends to contain a higher proportion of old mortar and exhibits greater water absorption [21]. It also contains a large amount of powder and interfacial transition zones [22]. As a result, concrete mixed with RFA tends to degrade more significantly than when an equivalent quantity of RCA is used [23]. The application scope for RFA is narrower than that of RCA, typically limited to low-strength concrete or mortar [21].

Current research reveals that the accelerated carbonation treatment of recycled fine aggregate can substantially enhance its quality, thereby improving the overall performance of the resultant concrete [24,25]. This approach also mitigates the ecological consequences linked to traditional aggregate extraction [26,27]. Despite many scholars focusing on the carbonation treatment of RCA over RFA, the potential benefits of treating RFA cannot be overlooked. The carbonation treatment method involves the diffusion and dissolution of CO_2_ in mortar pores attached to the surface of RFA. As a result, the calcium sources in the mortar become carbonated, resulting in the production of silica gel and calcium carbonate (CaCO_3_) crystals. These products improve the quality of RFA by filling the microscopic pores in attached mortar and microcracks [28,29,30]. Chinzorigt et al. [31] studied the effect of replacing recycled fine aggregate with 0–50% carbonized recycled fine aggregate on the compressive strength, carbonation resistance, and chloride ion penetration resistance of concrete and found that the compressive strength can be increased by up to about 15%, and the carbonation resistance and resistance to chloride ion penetration are improved. Previous studies also showed that the incorporation of carbonated recycled fine aggregate improved the density of recycled aggregate concrete and reduced the risk of corrosion of steel bars [32]. Furthermore, the effect of carbonation modification on the working performance of recycled fine aggregate concrete was studied in depth, and it was found that mortars prepared using carbonated recycled fine aggregate showed better fluidity and lower consistency loss values [33,34].

Existing research has demonstrated that the carbonation treatment significantly enhanced the physical properties of the treated RFA; specifically, it increased the apparent density while reducing the water absorption and crushing value in comparison to untreated RFA [35,36]. These improvements contribute to the effective performance of the recycled concrete, as evidenced by increases in both compressive strength and resistance to chloride ion penetration [32,36,37]. The previous studies have primarily concentrated on assessing the effects of accelerated carbonation on the performances of concrete containing either RCA or RFA, but these studies fall short in considering its effects on fully recycled aggregate concrete (FRAC). Therefore, this study examines the potential of accelerated carbonation to improve the properties of RFA. Furthermore, the FRAC, comprising 100% RCA and 100% RFA, was developed to assess the impact of carbonated RFA (C-RFA) at various replacement rates (0%, 25%, 50%, 75%, and 100%) on the compressive strength and durability (chloride penetration resistance and frost resistance) of FRAC.

## 2. Materials and Methodology

### 2.1. Materials

The RFA and RCA used in these experiments were produced using a process of crushing, cleaning, and sieving waste concrete supplied by Jiangsu Lvhe Environmental Technology Co., Ltd., located in Changzhou, China. The particle size range for RFA was between 0.16 mm and 4.75 mm, while the range for RCA spanned from 4.75 to 20 mm. The apparent density, water absorption, crushing value, and soundness of RCA were 2356 kg/m^3^, 4.6%, 15.4%, and 9.9%, respectively. The binding material employed to prepare concrete was Portland cement of grade 42.5 with an apparent density of 2963 kg/m^3^. Fly ash (FA), with an apparent density of 2759 kg/m^3^, and silica fume (SF), with an apparent density of 3067 kg/m^3^, were added as mineral admixtures. The content of FA and SF was 15% and 10% by weight of all cementitious materials, aimed at enhancing the workability, strength, and durability of FRAC. Table 1 illustrates the chemical compositions of various cementitious materials. To prepare RAC, additives such as a superplasticizer (water-reducing agent) and an air-entraining agent (AEA) were utilized. The dosage of superplasticizer was 0.5 wt.% of cementitious material to ensure that the workability of all FRAC could meet the slump value requirement of 150 mm.

### 2.2. Methodology

#### 2.2.1. Preparation of C-RFA and FRAC

The obtained RFAs were split into two portions: one served as the fine aggregates for the control group in FRAC; the other was designated to produce C-RFA. The transmission of carbon dioxide (CO_2_) from the cylinder toward the carbonation chamber occurred at 25 °C, 20 ± 2% concentration, 55 ± 5% relative humidity, and 0.5 MPa gas pressure. The rapid carbonation process was conducted over a period of 4 to 7 days, during which the RFA achieved a constant weight and exhibited no change in surface color upon phenolphthalein solution application, as reported by Liu et al. [9].

FRAC was composed of 100% RFA and 100% RAC. By replacing the RFA with C-RFA at different replacement rates (0, 25%, 50%, 75%, and 100%), the carbonated recycled fine concrete (C-RFC) was obtained, which was labeled C-RFC0, C-RFC25, C-RFC50, C-RFC75, and C-RFC100, respectively. The target strength of all concrete was 40 MPa, and the mix proportions are listed in Table 2. The concrete mixture was prepared using a two-stage mixing method. This method divides the mixing process into two parts and proportionally splits the required water into two parts, which are added after mixing one part with fine and coarse aggregate and cement, while the normal mixing approach only puts all the ingredients of concrete together and mixes them [14]. According to the previous studies, in the two-stage mixing approach, during the first stage of mixing, the use of half of the required water leads to the formation of a thin layer of cement slurry on the surface of RA, which permeates into the porous old cement mortar, filling up the old cracks and voids. To complete the cement hydration process, the remaining water is added during the second mixing stage, resulting in denser concrete, an improved interfacial zone around recycled aggregate, and, thus, a higher strength when compared with the traditional mixing approach [14,38].

During this study, initially, RCA and RFA were blended for 10–20 s. This was followed by the incorporation of half the water quantity, which was mixed for an additional 10–20 s. Subsequently, cement was introduced and combined for a duration of 50–60 s. Finally, the rest of the water and any admixtures were added, with the mixture being stirred for a further 50–60 s to ensure homogeneity. To enhance the workability and durability, mineral admixtures, such as fly ash (FA) and silica fume (SF), were incorporated. The frost resistance of the concrete was bolstered, and water content was minimized by introducing a combination of an AEA and a water-reducing agent. After the mixing process was completed, the fresh concrete mixture was transferred into molds, which were removed after 24 h. Subsequently, the specimens were placed in a standard curing chamber with a temperature of 20 ± 2 °C and relative humidity over 95% for a duration of 28 days.

**Figure 1:**
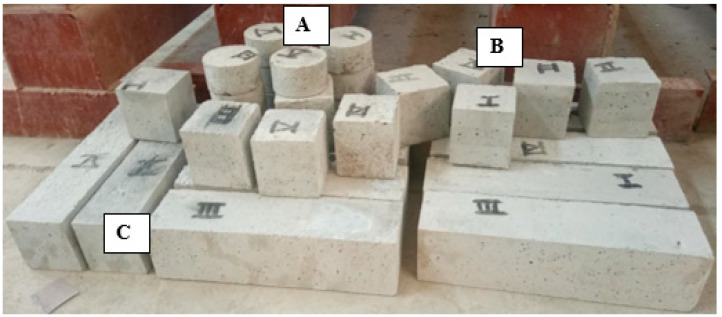
C-RFC labeled specimens: (**A**) Cylinder Φ 100 × 50 mm^3^; (**B**) Cubes 100 × 100 × 100 mm^3^; (**C**) Prisms 100 × 100 × 400 mm^3^.

The specimens designated for freeze–thaw (F-T) cycles and rapid chloride migration tests must be extracted 4 days earlier to conduct a preliminary saturation test. The dimensions of the specimens used for compressive strength, chloride penetration resistance, and frost resistance tests were 100 × 100 × 100 mm^3^, Φ 100 × 50 mm^3^, and 100 × 100 × 400 mm^3^, respectively (Figure 1).

#### 2.2.2. Measurements

##### Physical Properties of RFA and C-RFA

Based on the Chinese standard GB T14684-2011 [39], the physical properties of RFA and C-RFA, including apparent density, water absorption, and crushing value, were evaluated using a mass of 300 g.

##### Mechanical and Physical Properties of C-RFC

**Figure 2:**
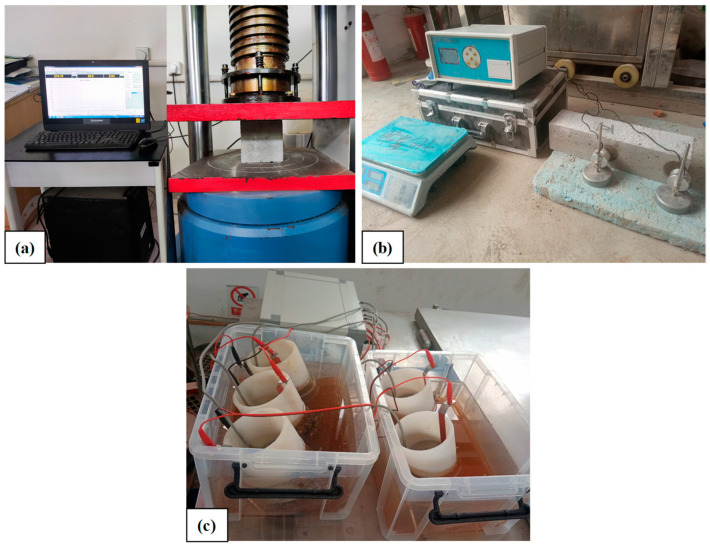
C-RFC testing: (**a**) Compressive strength; (**b**) Chloride ion penetration; (**c**) RDEM.

According to the Chinese standard GB/T 50081-2019 [40], the compressive strength of C-RFC with different contents of C-RFA was measured at the 28-day mark. Cubic specimens measuring 100 × 100 × 100 mm^3^ were examined using a hydraulic universal testing machine, as illustrated in Figure 2a. The average compressive strength was determined using three concrete cubes from each group. Additionally, the water absorption of concrete was calculated by Equation (1):

(1) $$W_{a}=\frac{m_{s}-m_{d}}{m_{d}}\times 100\%$$

where $W_{a}$ is the water absorption of concrete (%); $m_{s}$ is the mass of saturated surface dry state of water-saturated specimens (g); $m_{d}$ is the mass of dried specimens (g).

##### Chloride Ion Penetration

The rapid chloride migration coefficient method (RCM), in accordance with Chinese standard GB/T 50082-2009 [41], was used to evaluate the chloride penetration resistance of C-RFC cylindrical specimens (Figure 2b). The cathode solution used in this test was 10% NaCl solution, and the anode solution was 0.3 mol/L NaOH solution. After the chloride ion penetration was completed, the specimens were cut to measure the penetration depth. The AgNO_3_ standard solution with a concentration of 0.1 mol/L was used as an indicator, and the rapid chloride ion migration coefficient was calculated by Equation (2):

(2) $$D_{\mathrm{RCM}}=\frac{0.0239\times (273+T)L}{(U-2)t}\left(X_{d}-0.0238\sqrt{\frac{(273+T)LX_{d}}{U-2}}\right)$$

where $D_{\mathrm{RCM}}$ is the rapid chloride ion migration coefficient (0.1 × 10^−12^ m^2^/s); U is the applied voltage (V); T is the average value of the initial and final temperatures of anodic solution (°C); L is the thickness of specimen (mm); $X_{d}$ is the average depth of chloride ion penetration (mm); t is the test duration (h).

##### Frost Resistance

As shown in Figure 2c, the relative dynamic elastic modulus (RDEM) and mass loss of C-RFC specimens were recorded before and after subjecting to F-T cycles [42]. Measurements were conducted every 50 cycles up to a total of 300 cycles, in accordance with Chinese standard GB/T 50082-2009 [41]. The transverse fundamental frequency and mass of the specimens were measured every 50 F-T cycles, and the relative dynamic elastic modulus (RDEM) and mass loss rate (MLR) were calculated separately by Equations (3) and (4) to assess the frost resistance of the specimens:

(3) $$RDEM_{n}=\frac{f_{n}^{2}}{f_{0}^{2}}\times 100\%$$

(4) $$MLR_{n}=\frac{m_{0}-m_{n}}{m_{0}}\times 100\%$$

where $RDEM_{n}$ is the RDEM after n F-T cycles (%; $MLR_{n}$ is the mass loss rate after n F-T cycles (%); $f_{n}$ is the transverse fundamental frequency after n F-T cycles (Hz); $m_{n}$ is the mass after n F-T cycles (kg); $f_{0}$ is the initial transverse fundamental frequency (Hz), and $m_{0}$ is the initial mass (kg).

##### Microscopic Analysis of C-RFC

The microstructure features of the concrete prepared with C-RFA were analyzed using a digital Vickers Microhardness Tester (HVS-1000SS, GOYOJO, Shenzhen, China). Elements such as ITZs, pores, and cracks within the C-RFC were examined using the Scanning Electron Microscopy (SEM) images generated for each group.

## 3. Results and Discussion

### 3.1. Physical Properties of RFA and C-RFA

This study evaluated the physical properties of RFA before and after undergoing carbonation treatment. The detailed results are systematically cataloged in Table 3. The recorded apparent density of RFA and C-RFA were 2476 kg/m^3^ and 2577 kg/m^3^, respectively, marking a rise of 4.1%. The water absorption displayed a reduction from 8.8% to 6.9%, while the crushing value exhibited a marked reduction, dropping from 25.7% to 19.8%. Namely, the water absorption and crushing value exhibited a decrease of 21.6% and 22.9%, respectively. This result signified an improvement in the quality classification of RFA from Grade III to Grade I, as per the standards outlined in GB/T 25176-2010 [43]. These research findings corroborated the global scholarly consensus that CO_2_ carbonation enhanced the apparent density of RCA, with a notable uptick observed as the particle size of the RCA decreased [44,45,46].

**Figure 3:**
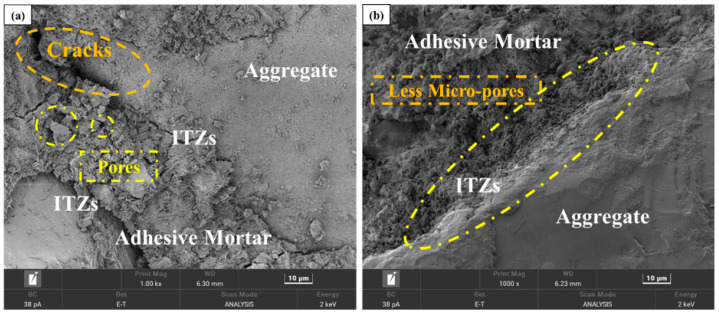
Micro-structure of RFA (**a**) and C-RFA (**b**).

The observed reduction in the decrease in water absorption and crushing value of C-RFA is attributed to the formation of calcium carbonate (CaCO_3_) particles [47]. These substances are produced by the reaction between the hydration products of attached mortar in RFA and CO_2_. The attached mortar coating of the RFA underwent carbonation treatment, yielding calcium carbonate (CaCO_3_) particles. These particles bridged the gaps and fissures within the old mortar, fortifying the ITZ between it and the original aggregates (Figure 3), thus yielding a more consolidated aggregate framework [28]. As a result, the structure and quality of C-RFA were significantly improved.

### 3.2. Physical and Mechanical Properties of C-RFC

#### 3.2.1. Water Absorption

**Figure 4:**
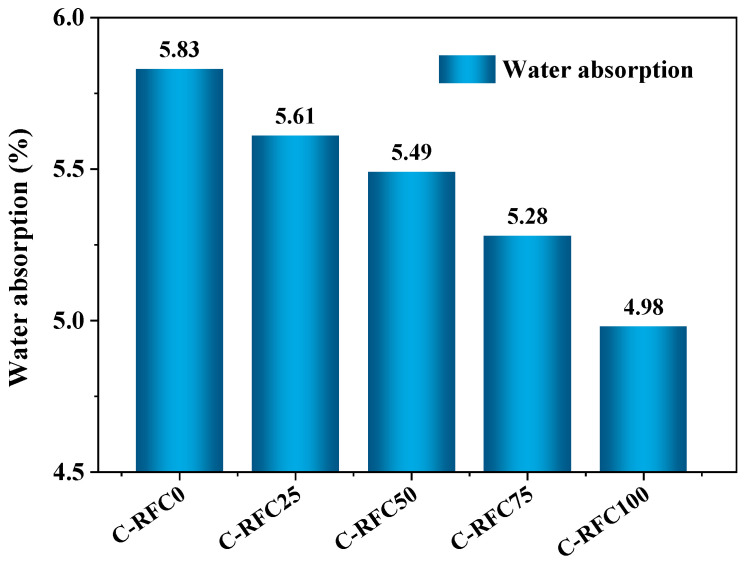
Water absorption of C-RFC with different contents of C-RFA.

Figure 4 depicts the correlation between water absorption of RFC and different C-RFA replacement rates. With the escalating replacement rate of C-RFA, a corresponding gradual decline in the water absorption of the RFC was observed, diminishing from 5.83% in C-RFC0 to 4.98% in C-RFC100. The water absorption capacity of concrete is significantly affected by its pore structure [9,48,49,50]. The results presented above demonstrated that incorporating C-RFA into recycled concrete could positively modify the pore structure of the concrete. This confirmed that the rapid carbonation process applied to RFA was an effective method for reducing water absorption and decreasing the overall porosity in C-RFC.

#### 3.2.2. Compressive Strength

**Figure 5:**
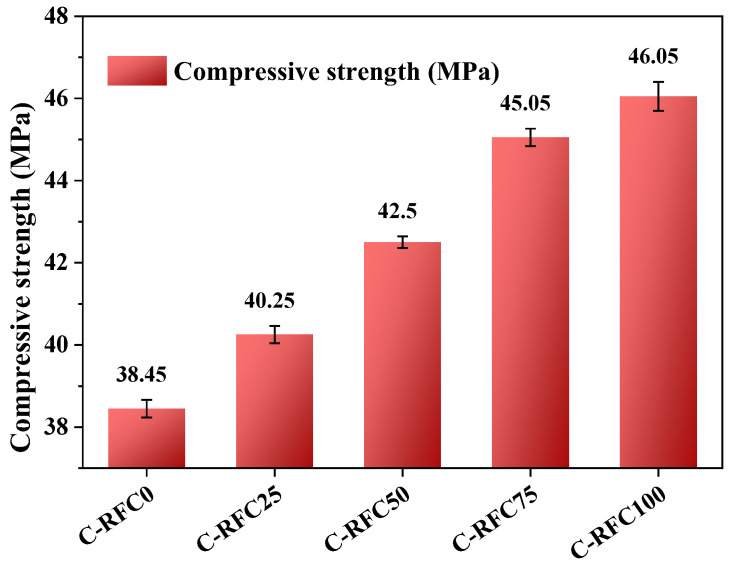
Compressive strength of C-RFC.

Figure 5 presents the variation in compressive strength of C-RFC corresponding to different replacement rates of C-RFA. It was observed that the FRAC, that is, the concrete with a C-RFA replacement rate of 0% marked as C-RFC0, exhibited a compressive strength of merely 38.45 MPa, falling short of the prescribed design criteria. But, as the replacement rate of C-RFA rose, a consistent enhancement in the compressive strength of concrete was recorded, echoing observations made in earlier research [9,51]. When RFA was entirely replaced with C-RFA in the FRAC mixture, the compressive strength of C-RFC notably increased, reaching a value of 46.8 MPa. This was comparable to the compressive strength of natural aggregate concrete after a curing period of 28 days [52,53].

With an increase in the replacement rate of C-RFA, compressive strength shows a linear increase. The compressive strength of C-RFC100 marked a significant increase of 19.8% relative to the initial strength of baseline C-RFC0. After accelerated carbonation treatment, the apparent density of RFA markedly increased, while the water absorption and crushing value decreased substantially, indicating a denser structure of CRFA [54]. Such density contributed to a more consolidated concrete matrix and a reinforced interfacial transition zone (ITZ) between the old mortar of the RFA and the new mortar in concrete, ultimately leading to an increase in compressive strength [51,55].

### 3.3. Durability of C-RFC

#### 3.3.1. Chloride Ion Permeability of C-RFC

**Figure 6:**
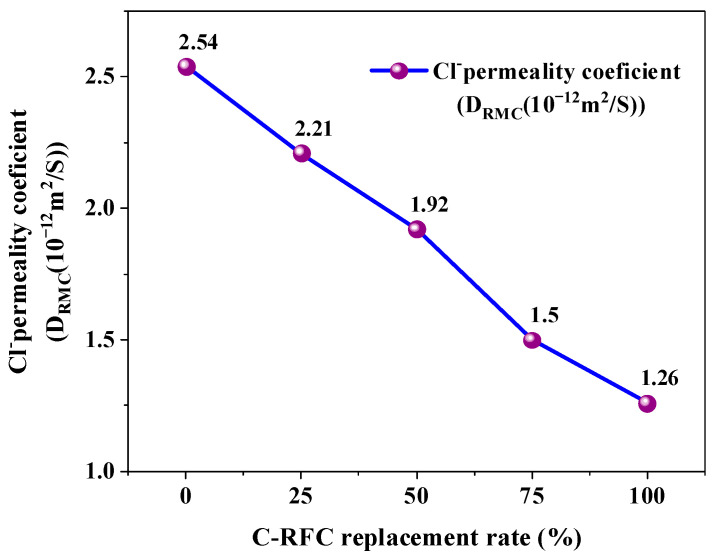
Chloride ion permeability coefficient of C-RFC.

Chloride ion ingress often leads to corrosion of steel within reinforced concrete, thereby reducing the structure’s safety and longevity [56,57]. Due to the high porosity of recycled aggregate, the recycled concrete is more vulnerable to chloride ion intrusion. However, the carbonation treatment improved the pore structure of the recycled aggregate, thereby increasing its resistance to chloride ion penetration. Figure 6 illustrates the chloride ion permeability coefficient of C-RFC. As the replacement rate of C-RFA increased from 0% to 100%, the chloride ion permeability coefficient progressively decreased from 2.54 × 10^−12^ m^2^/s to 1.26 × 10^−12^ m^2^/s, respectively, representing a decrease of 50.4%. It is worth noting that even for FRAC without carbonated aggregates, the chloride ion permeability coefficient was lower than 4 × 10^−12^ m^2^/s, meeting the requirements for Type E chloride exposure environments for a concrete structure with a service life of 100 years according to the standard of GB/T 50476–2019 [58].

#### 3.3.2. Frost Resistance

**Figure 7:**
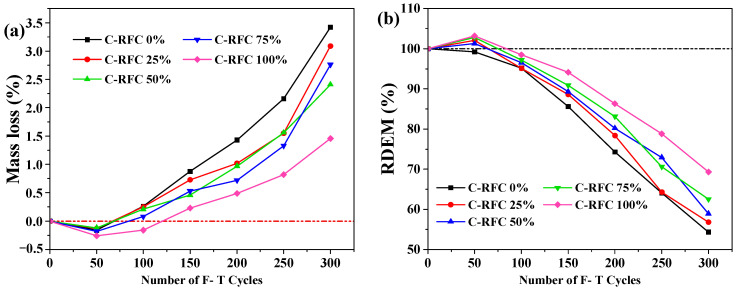
(**a**) Mass loss rate of C-RFC; (**b**) RDEM of C-RFC under F-T Cycles.

In a manner akin to enhancing chloride ion permeability resistance, the incorporation of C-RFA was found to bolster the frost resistance of C-RFC. Figure 7 shows the mass loss and RDEM of C-RFC for each group after subjecting to F-T cycles.

Under the influence of fluctuating temperatures, the degradation of C-RFC exhibited a non-linear pattern of mass change, characterized by an initial increase followed by a decrease (Figure 7a). Throughout the recurring freeze–thaw cycles in concrete, the volumetric expansion induced by the freezing of entrapped water generates frost heave pressure. Simultaneously, the migration of supercooled water toward the ice boundary results in osmotic pressure. These combined forces instigate the progressive enlargement of microcracks within the concrete [59]. When a significant number of these cracks interconnect, they create a web-like fracture network, potentially leading to surface erosion or the outward diffusion of the internal fracture network, culminating in surface cracking of the concrete [59]. This process intensifies the deterioration of the concrete, precipitating a decline in mass and even a corresponding reduction in strength. During the early stages of F-T cycles, the frost heave and osmotic pressures induced the emergence of micro-cracks, which, however, did not yet lead to the detachment of the concrete’s surface mortar. This condition elevated the concrete’s capacity to absorb water, thereby leading to an incremental gain in its mass. But with the progressive intensification of the freeze–thaw cycles, the resultant damage escalated, culminating in a steady amplification of mass loss.

Surprisingly, the mass loss of C-RFC with C-RFA was consistently lower than that of C-RFC0, with the difference being particularly notable in the case of C-RFC100.

After 300 F-T cycles, the mass loss rate of C-RFC100 was only 1.46%. The concrete from the other groups also satisfied the requirement of a mass loss of less than 5%, thus not reaching the failure criterion [41]. Figure 7b depicts the variations in the RDEM across different replacement rates as influenced by the number of freeze–thaw cycles. Similar to the mass loss results, the incorporation of C-RFA enhanced the RDEM of C-RFC. But following 300 freeze–thaw cycles, only the RDEM of CRFC75 and CRFC100 satisfied the operational standard. The concrete specimens with other contents of C-RFA fell below 60%, reaching the failure criterion.

### 3.4. Microscopic Analysis of C-RFC

**Figure 8:**
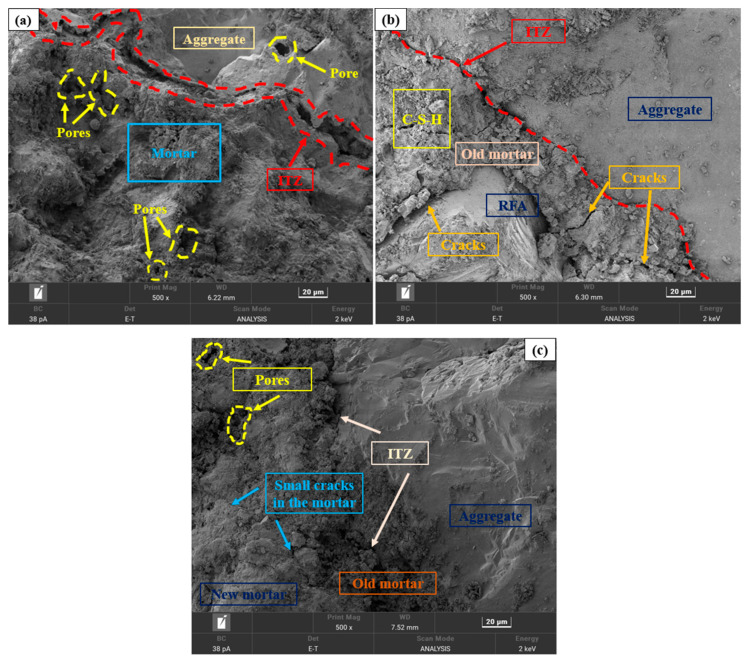

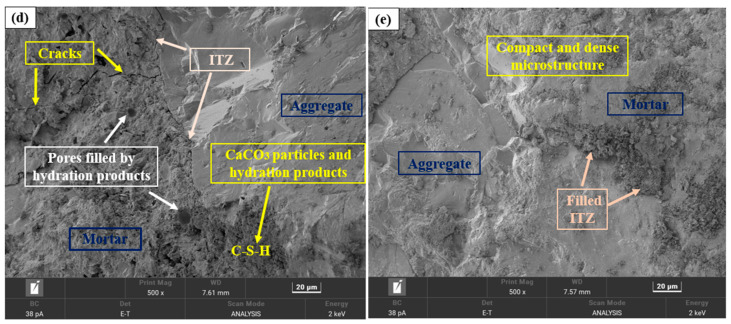
SEM images of C-RFC: (**a**) C-RFC0; (**b**) C-RFC25; (**c**) C-RFC50; (**d**) C-RFC75; (**e**) C-RFC100.

The SEM images featured in Figure 8 portray the microstructural integrity of C-RFC at different C-RFA replacement rates. The findings showed that the pore structure in mortar, the ITZ width, and the number of micro-cracks in C-RFC were proportional to the level of C-RFA content.

As illustrated in Figure 8a, the microstructure of C-RFC0 exhibited relative weakness, characterized by numerous large pores within the attached mortar, pronounced cracks, and wider ITZ. This structural compromise accounted for the suboptimal compressive strength and diminished resistance to chloride permeability of FRAC. However, with the increase in C-RFA content, the ITZ between the attached mortar coated on the surface of CRFA and the new concrete mortar narrowed, accompanied by a decline in micro-cracks and voids, resulting in a denser overall structure. This compaction was ascribed to the CaCO_3_ particles formed on the RFA from carbonation, which coated and reinforced the interface. Given that CaCO_3_ possessed greater hardness than the hydration products in the old mortar, it substantially elevated the quality of C-RFA [60,61].

Additionally, these fine CaCO_3_ particles served as nucleation sites, promoting a significant formation and growth of the C-S-H gel on the C-RFA’s surface, which, in turn, seamlessly filled the ITZ and existing voids, enhancing the concrete’s structure [62]. Surprisingly, the ITZ between the old mortar and the new mortar was greatly mitigated or even eliminated, particularly in CRFC100, where this ITZ was nearly imperceptible, as shown in Figure 8e. This indicated that carbonation-modified RFA could significantly enhance the performance of FRAC.

## 4. Conclusions

A comprehensive experimental investigation into the impact of carbonated recycled fine aggregates on the mechanical properties and durability of fully recycled aggregate concrete was conducted in this work. From the experimental data and subsequent analysis, we have arrived at the following conclusions:

(1) In comparison to untreated RFA, the apparent density of C-RFA enhanced by rapid carbonation treatment saw an increase of 4.1%. Concurrently, the 24-h water absorption and crushing values decreased by 21.6% and 22.9%, respectively. As a result of carbonation modification, the RFA was transformed from a Grade III material to a superior Grade I, affirming its enhanced suitability for use in concrete construction;
(2) The carbonated recycled fine aggregate plays a positive role in concrete production. The compressive strength of C-RFC experienced a positive trajectory as the C-RFA content increased; with a 25% substitution rate, the strength achieved 40.7 MPa, exceeding the target threshold of 40 MPa. At full replacement, the strength further escalated to 46.05 MPa. This trend highlighted the potential of C-RFA to enhance the microstructure of recycled aggregate concrete (RAC), offering a proven approach to decrease water absorption and porosity, thereby augmenting the compressive strength of FRAC;
(3) The incorporation of C-RFA into FRAC led to a discernible enhancement in its resistance to the rigors of freeze–thaw cycles. Upon reaching a 100% C-RFA mixture, a significant reduction of about 50.4% in the chloride permeability coefficients was observed; FRAC can satisfy the requirements of the standard for Type E chloride exposure environments for concrete structures with a service life of 100 years;
(4) With the increase in C-RFA content, the frost resistance durability of FRAC was improved significantly. Across a substitution spectrum from 25% to 100% for C-RFA, C-RFC demonstrated a mass loss rate below 3.0% after withstanding 300 freeze–thaw cycles. Furthermore, as the number of freeze–thaw cycles increased, the RDEM of FRAC with 0~50% C-RFA content exceeded the 60% threshold. The frost resistance durability of FRAC was unable to meet the requirements of 300 freeze–thaw cycles;
(5) Carbonation treatment served to densify the mortar structure attached to the surface of the RFA and strengthened the ITZ between the old mortar and the original aggregates, consequently enhancing the quality of the RFA. The CaCO_3_ particles encrusting the RFA surface were characterized by their considerable hardness and acted as inducers for the generation of cement hydration products, thus consolidating the porosity and ITZ between RFA and new mortar in the FRAC.

Based on the research findings, FRAC prepared with carbonation-modified RFA exhibits excellent performance in both mechanical and durability aspects. This provides a positive significance for the advancement of fully recycled aggregate concrete.

## 5. Limitations and Future Works

Currently, governments worldwide are actively striving to achieve the goals of “peak carbon emissions and carbon neutrality”. Utilizing construction and demolition wastes to prepare recycled concrete undoubtedly constitutes a crucial measure for energy conservation and emission reduction in the construction industry. This study explores the enhancement effects of carbonation-modified treatment on the quality of recycled fine aggregate and the application potential of fully recycled aggregate concrete, which contributes to further improving the utilization rate of waste concrete. However, it is worth noting that due to the particularity of recycled aggregate, the long-term performance of fully recycled aggregate concrete, including creep and crack resistance, warrants further in-depth research.

Our future work will focus on the long-term performance of fully recycled concrete, such as drying shrinkage and autogenous shrinkage, as well as the enhancement effects of different aggregate modification methods on the performance of fully recycled aggregate concrete. Additionally, after fully recycled aggregate concrete reaches failure or the design service life, its potential for regeneration will be investigated in future studies.

## Figures and Tables

**Table 1:** Chemical compositions of cementitious materials (wt.%).

Component	CaO	SiO_2_	Al_2_O_3_	Fe_2_O_3_	MgO	MnO	K_2_O	TiO_2_	SO_3_
Cement	61.01	20.41	7.42	3.74	1.26	0.15	0.75	0.28	2.07
Silica Fume (SF)	0.23	86.18	1.08	0.93	0.78	0.12	--	--	0.84
Fly Ash (FA)	3.82	52.5	28.33	3.67	1.12	0.20	1.69	0.97	1.75

**Table 2:** Mix proportions of C-RFC (kg/m^3^).

C-RFA (%)	RCA	RFA	C-RFA	Cement	Fly Ash	Silica Fume	Water	Water Reducing Agent	Air Entraining Agent
C-RFC0	906	658	0	319	64	43	166	2.13	0.13
C-RFC25	906	493	171	319	64	43	166	2.13	0.13
C-RFC50	906	329	342	319	64	43	166	2.13	0.13
C-RFC75	906	164	513	319	64	43	166	2.13	0.13
C-RFC100	906	0	685	319	64	43	166	2.13	0.13

**Table 3:** Physical properties of RFA and C-RFA.

Name	Apparent Density (kg/m^3^)	Water Absorption (%)	Crushing Value (%)
RFA	2476	8.8	25.7
C-RFA	2577	6.9	19.8

## Data Availability

Data are contained within the article.

## Nomenclature

FRAC	Fully recycled aggregate concrete
C-RFA	Carbonated recycled fine aggregate
C-RFC	Carbonated recycled fine aggregate concrete
C&DW	Construction and demolition waste
NCA	Natural coarse aggregate
FA	Fly ash
SF	Silica fume
F-T cycles	Freeze-thaw cycles
RCM	Rapid chloride migration
RDEM	Relative dynamic elastic modulus
ITZs	Interface transition zones
AEA	Air-entraining agent
